# Challenges and Solutions for Leave-One-Out Biosensor Design in the Context of a Rugged Fitness Landscape

**DOI:** 10.3390/s24196380

**Published:** 2024-10-01

**Authors:** Shounak Banerjee, Keith Fraser, Donna E. Crone, Jinal C. Patel, Sarah E. Bondos, Christopher Bystroff

**Affiliations:** 1 Los Alamos National Laboratory, Los Alamos, NM 87545, USA; baners4@lanl.gov; 2 Biological Sciences, Rensselaer Polytechnic Institute, Troy, NY 12180, USA; frasek2@rpi.edu (K.F.); croned@rpi.edu (D.E.C.); jcp353@cornell.edu (J.C.P.); 3 Medical Physiology, Texas A&M University, College Station, TX 77843, USA; bondos@tamu.edu; 4 Computer Science, Rensselaer Polytechnic Institute, Troy, NY 12180, USA

**Keywords:** green fluorescent protein, computational design, chromophore, ultrabithorax fibers

## Abstract

The leave-one-out (LOO) green fluorescent protein (GFP) approach to biosensor design combines computational protein design with split protein reconstitution. LOO-GFPs reversibly fold and gain fluorescence upon encountering the target peptide, which can be redefined by computational design of the LOO site. Such an approach can be used to create reusable biosensors for the early detection of emerging biological threats. Enlightening biophysical inferences for nine LOO-GFP biosensor libraries are presented, with target sequences from dengue, influenza, or HIV, replacing beta strands 7, 8, or 11. An initially low hit rate was traced to components of the energy function, manifesting in the over-rewarding of over-tight side chain packing. Also, screening by colony picking required a low library complexity, but designing a biosensor against a peptide of at least 12 residues requires a high-complexity library. This double-bind was solved using a “piecemeal” iterative design strategy. Also, designed LOO-GFPs fluoresced in the unbound state due to unwanted dimerization, but this was solved by fusing a fully functional prototype LOO-GFP to a fiber-forming protein, *Drosophila* ultrabithorax, creating a biosensor fiber. One influenza hemagglutinin biosensor is characterized here in detail, showing a shifted excitation/emission spectrum, a micromolar affinity for the target peptide, and an unexpected photo-switching ability.

## 1. Introduction

In the absence of effective vaccines for viral diseases, early detection represents the most effective strategy for mitigating disease spread. Insect-borne viruses provide an easily collected detection target where the virus resides in high concentration. If we envision large-scale viral detection systems on mosquito traps or in sewer lines [1], economic concerns force us to avoid the reagents and equipment needed for *in situ* PCR-based monitoring [2]. An alternative is to detect virus-specific proteins using a computationally designed fluorescent biosensor, which requires simple reagents, can be repeatedly reused by unfolding and refolding, and can be monitored *in situ* using a blue light and a camera.

**Figure 1:**
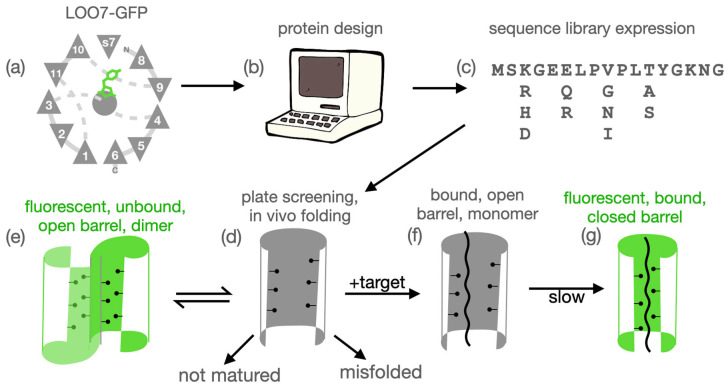
Overview. (**a**) TOPS [13] diagram of LOO7-GFP with bound target peptide s7 replacing beta strand 7. (**b**) Computational protein design of the surrounding residues using Rosetta or DEEdesign accommodates the target peptide. (**c**) Design followed by simulated melting generates a sequence expression of desired complexity. The physical library is synthesized by degenerate-oligonucleotide assembly PCR and transformed into bacteria. (**d**) Individual clones co-expressed with target may fold. Or, they may misfold and/or not mature. (**e**) Correctly folded, unbound LOO-GFP, an open barrel, may form a dimeric state which matures and fluoresces. (**f**) Binding of target peptide shifts equilibrium to the bound state. (**g**) Target-bound LOO-GFP may or may not undergo barrel closure, the kinetic slow step, leading to maturation/fluorescence.

With this aim we have developed leave-one-out green fluorescent protein (LOO-GFP), a platform for protein design that enables sequence-specific binding to short peptides producing a fluorescent signal [3,4,5]. The platform (Figure 1) was inspired by many known instances of splitting and reconstituting protein structure and function [6,7,8,9,10,11]. LOO-GFP is green fluorescent protein that has been circularly permuted and split to remove one of the eleven beta strands that make up the GFP barrel. There are eleven possible LOO-GFPs. Six of these (removal of beta strands 4, 5, 7, 8, 9, 10, or 11, see Figure 1a) are known to reconstitute fluorescence in vivo [12]. These are denoted LOO*n*-<target>, where *n* is the number of the strand left out and <target> is the source of the target sequence (or “GFP” if the target is the wild-type left-out strand). LOO*n*-<target> is also used to refer to a library of LOO*n* sequences designed and screened against <target>. LOO*n*-<target>:X is a specific clone from that library, and LOO*n*-<target>:X•Y is that protein bound to peptide Y. Algorithms and tools for designing the empty strand site to specifically accept a different exogenous peptide sequence have been developed [4], along with high throughput screening methods for identifying good biosensor sequences in a library [4].

We have succeeded in computationally designing a LOO7-GFP to recognize a specific sequence from H5N1 influenza (Thailand 2004 strain) haemagglutinin (HA) [4]. When co-expressed with a 12-residue HA peptide (SSHEVSLGVSSA) in *E. coli*, a library of biosensor sequences (LOO7-HA4) produced dozens of fluorescent clones, and selected clones were studied in detail. But the in vitro complementation of clone LOO7-HA4:DS2 and the peptide HA revealed a surprising drop in fluorescence, the opposite of the expected outcome [1]. This drop was accompanied by a transition from dimer to a monomer. To our surprise, the dimeric unbound biosensor was fluorescent but the peptide-bound monomer was not! These results and challenges set the stage for the current work.

Immobilization of LOO-GFP should prevent dimerization and its consequent unbound autofluorescence. To test this hypothesis, we fused LOO-GFPs N-terminally to *Drosophila* ultrabithorax (Ubx), which forms fibers through dityrosine bonds [14]. LOO8-GFP fused to Ubx (LOO8-GFP-Ubx) forms fibers identical to unadulterated Ubx and fluoresces when co-expressed with GFP strand 8 (s8) [15]. Background fluorescence was observed in the polymerized fibers but was eliminated by diluting LOO8-GFP-Ubx 1:10 with wild-type Ubx. However, to avoid a permanent loss of fluorescence upon refolding, we needed to add 1.25 M sodium chloride, presumably to mask the highly charged Ubx domain. The result was reusable and programmable biosensor fibers [15]. In the fiber construct reported here, the highly charged Ubx is blocked by a bulky spacer, maltose-binding protein (MBP), in between the LOO-GFP and Ubx domains. This construct successfully recovers fluorescence in the presence of its peptide, and not in its absence, when the biosensor is regenerated by refolding.

Having eliminated all false positives of dimerization, we nonetheless found a very low rate of *true* positives in library screens. Our *native* format library screens described herein showed the target peptide fused genetically to the biosensor, therefore the biosensor cannot exist in the unbound state and cannot dimerize. In the *native* screen all fluorescent colonies are true positives. But we saw very few fluorescent colonies, even when the library complexity was low enough to screen efficiently (1000–3000 sequences). We traced the problem to a combination of a prohibitively large design space (28 to 38 positions were designable) and a rough energy landscape of side chain packing in the core of the barrel. The rough energy landscape means that there is a “domino effect” in side chain packing, making the design space highly covariant and difficult to sample. Reducing the number of designable positions to 7 to 13 positions reproducibly produced fluorescent colonies (Table 1), but this required using a “*piecemeal*” strategy to design the complete peptide binding site in pieces [16].

Confusingly, tight packing in protein design has been variously credited for rigidity and thermostability [17,18,19,20,21,22], but also cited for loss of native-like structure [23]. Perhaps the outcome depends on the protein architecture. GFP is a beta barrel protein with a fixed and immutable contained volume. As such, it may be more sensitive than other architectures to overpacking in the core as a result of designed mutations [24,25,26]. In our analysis of the library screening results from a structural perspective, we attribute the low hit rate to the tight packing.

Acting on the hypothesis that overpacking led to false positives, we designed a new influenza biosensor library called LOO7-HA5. Rational choices were made to the design palette to reduce overpacking. As intended, the library produced a sequence showing a gain in fluorescence upon binding. The increased QY is shown to be the result of a closed, native-like barrel.

These experiments, the challenges, and their solutions have broadened our understanding of the design space of GFP, painting a picture of a rough fitness landscape of design [25,27], and leading to a new strategy for design—a fiber-based method for LOO-GFP biosensor library screening—and a piecemeal approach for iterative sampling of the design space.

Currently, the detection of sequence-specific peptides within proteins is performed by antibody-based methods, such as Western blot or dot plot. This requires denaturing and immobilizing the analyte and, of course, raising antibodies to the peptide. The advantage of our method is that no antibodies need to be raised and that the binding produces an intrinsic signal, green fluorescence.

## 2. Materials and Methods

### 2.1. Target Selection

Target peptides were chosen based on sequence similarity to pertinent LOO strands. A manually curated structure-based sequence alignment of all fluorescent proteins with known structures (ca. 2012) was used to create a PROSITE [28] format regular expression for input to an in-house pattern search tool. Square brackets [] indicate allowed, curly brackets {} indicate disallowed. Sequences that matched the pattern were further excluded to avoid N-glycosylation sites, where applicable.

### 2.2. Computational Protein Library Design

In this discussion, “*design space*” refers to the number of residue positions that are variable, while “*design palette*” refers to set of amino acids at each position of a *design space*. In computational protein design, the input is a *design palette* and the output is a “*library*”, which is then encoded using degenerate codons, and then screened experimentally. “*Complexity*” is the number of sequence permutations encoded in the library. The “*template*” consists of backbone atoms and all non-designed side chain atoms, including chromophore and waters. Residue numbering for all sequences discussed here is taken from superfolder GFP (sfGFP, PDB:2B3P), regardless of any truncations, extensions, or circular permutations.

The LOO7-HA4 library was calculated using DEEdesign as previously described [4]. The input design palette was set by mutating the LOO strand to the target sequence, then creating the palette of each nearest neighbor side chain manually by inspection of the structure in the MOE molecular modeling suite (Chemical Computing Group, Montreal, QC, Canada) and choosing amino acids. The palette and the template are input to the design program DEEdesign. The palette (or library) is encoded as a plain-text character string with brackets for designed residues. Within DEEdesign, the library expression was narrowed to the desired complexity using the Dead-end Elimination (DEE) algorithm [29] followed by Simulated Melting (SM) [4]. Table 1 shows the output libraries.

All other libraries were calculated using the Rosetta Macromolecular Modeling suite (Rosetta) [30,31] using a parallel computing script called “*plastic protein design*” (PPD) [32]. PPD attempts to capture the dynamics of backbone and side chain atoms during design. To start, we generate a template ensemble using the Rosetta Backrub (BR) tool [33], where parameters were tuned as described previously [34]. Then, in parallel calculations, individual compute nodes proceeded with Rosetta protein design independently, using the Metropolis Monte Carlo algorithm to select side chain rotamers from the Dunbrack backbone-dependent rotamer library [35] subject to the *talaris2013* scoring function [31]. In PPD, compute nodes share their progress with each other periodically. If a side chain rotamer is accepted by all the members of the template ensemble then it is kept, even if each ensemble member accepts a slightly different conformation, called a *sub-rotamer*. This method was meant to select for side chain rotamers that move in concert with the backbone. The improvement or lack thereof of the PPD approach as compared to static template methods is difficult to experimentally verify, but several protein designers tout the importance of modeling motion when designing proteins [36,37,38].

Rational redesign of the LOO7-HA5 library was performed using MOE to evaluate a library that was generated as follows. Briefly, homology models of LOO7-HA4 [4] in the pre- and post-chromophore maturation states were generated using Rosetta, with the Cycle3 R96M mutant (PDB:2AWJ) as the template for the pre-maturation state and superfolder GFP (PDB: 2B3P) as the template for the post-maturation state. Background mutations were simulated for transitions to the superfolder OPT [8] template. For both models, iterations of mutation and side chain packing were performed in torsion space, using the Rosetta PackRotamersMover function and subsequent energy minimization using the MinMover function, both using the *talaris2013* scoring function [31]. This protocol was used for subsequent determination of minimum energy configurations (MECs). Rotamers compatible with both states of the protein were selected manually. Starting from the MEC, SM was performed to generate libraries of the desired complexity. Buried waters were added to MECs using DOWSER [39].

Manual intervention in library design was carried out as follows. The design selections after SM were compared to a manually curated sequence profile representing several GFP homologs, based on structural alignment. The library was pruned or otherwise modified based on visual assessment of side chain’s environment in MOE, along with the presence or absence of the amino acid in the sequence profile of fluorescent proteins, while considering the placement of internal waters. The resulting library (LOO7-HA5) after computational and manual design before experimental screening had a complexity of 276,480 sequences.

“*Piecemeal protein design*” is a strategy to reduce the design space, thereby increasing the likelihood of success in finding a fluorescent colony (*hit*). The target, usually a 12–14-residue peptide, is divided into pieces which may be as small as 2 residues. Each piece is used to define a design space as above. After computational design, experimental screening, and sequence analysis of the *hits*, neighboring pieces are joined, and the *hit* sequences are used to define the design palette for another round of experimental screening (no additional computational screening was performed). The screened libraries of the pieces were coalesced to make a library for the full length of the target peptide.

### 2.3. Library Construction and Plate Screening

Gene libraries were generated by encoding all mutations from the computationally designed library from DEEdesign or Rosetta at each position with degenerate codons, then preparing the gene for assembly PCR using DNAworks [40]. The output of DNAworks are the sequences of overlapping, single-stranded, forward and reverse 60-base oligonucleotides optimized for a 60 °C annealing temperature. Assembly PCR [41] was performed to link all overlapping oligos, and in doing so incorporating variable codons randomly. Inclusion bias at this step was tested for as described in the Supplementary Data and found to be absent. The first forward oligo contains an NcoI site and the last reverse oligo an EcoRI site. To make the oligo pool, 1 picomole of each oligo was added to a standard 50 μL Phusion DNA polymerase PCR mixture (New England Biolabs, Ipswich, MA, USA). Twenty-five cycles of amplification were performed at an annealing temperature of 60 °C and with an extension time of 1 min. One microliter of the product from assembly PCR was used as the template for standard amplification PCR using the terminal oligonucleotides as primers.

Most of the libraries were expressed in the “*native*” library format with the target sequence replacing the LOO segment and without circular permutation. The rest of the gene outside of the target sequence contained the designed mutations as degenerate codons. The purified DNA library was cut with NcoI and EcoRI and cloned into the pET28a+ vector for expression in BL21(DE3) *E. coli*. Transformed bacteria were plated on nitrocellulose membranes over selective media. Protein expression was then induced by transferring membranes to plates containing 0.5 mM IPTG and antibiotics. Fluorescent colonies were grown up and sequenced (MCLAB, S. San Francisco, CA, USA). Table 1 shows the libraries, with highlighting (black background) for *hits*, meaning amino acids found in the fluorescent colonies, if any. The sequences were modeled onto the template structure and inspected using MOE.

Libraries LOO7-HA4, LOO7-HA5, and LOO7-EDIII were screened in *leave-one-out* (LOO) format, constructed by permuting and truncating the coding sequence to remove the target strand. A glycine-rich linker (GGSGGT) was used to bridge the original N- and C-termini. A degenerate-codon-containing oligonucleotide set, with 5’ NcoI and 3’ EcoRI restriction sites, was used to assemble each LOO library. After cutting with NcoI/EcoRI this library was inserted into the pCDFDuet-1 vector at multiple cloning site 1. The target peptide was fused C-terminally to *Synechocystis* sp. intein, as described previously [42], and was inserted into multiple cloning site 2. Constructs were confirmed by restriction digest and sequencing.

LOO7-EDIII and LOO8-NS1#4 were screened in the *leave-it-in* (LII) format. Termini were linked as for LOO format, where the target peptide served as the new N-terminus. The purified DNA library was cut with NcoI and EcoRI and cloned into the pET28a+ vector for expression in BL21 E. coli. Plate screening was performed as for the native libraries. If a library was screened in more than one format, then *native* format was screened first, followed by LII, followed by LOO.

### 2.4. LOO7-HA5:ES1 Expression and Purification

One colony of LOO7-HA5, called “ES1”, was selected for detailed analysis. LOO7-HA5:ES1 was expressed in the target peptide-bound state and purified, as previously described for LOO7-HA4 [4]. The only exception is that expression was induced at 18 °C instead of 37 °C. The purified and dialyzed biosensor–peptide complex LOO7-HA5:ES1•HA5 was buffer exchanged into denaturing buffer containing 6 M guanidinium hydrochloride (GndHCl) and incubated at room temperature for 2 h to remove the HA5 peptide. The denatured protein solution was passed through a 1 cm column of pre-equilibrated *HisPur* resin (Thermo Scientific, Waltham, MA, USA) by gravity flow. Three column volumes of denaturing buffer were used to wash away any remaining peptide. Slow refolding was then performed by using a gradient to gradually remove GndHCl. This was followed by the addition of 6 mL of elution buffer to elute the protein from the column. The refolded LOO protein, now devoid of the “priming” target peptide, was dialyzed into a buffer containing 10 mM sodium phosphate and 5 mM sodium chloride at pH 8.0 overnight at 4 °C.

### 2.5. LOO7-HA5:ES1 Size Exclusion Chromatography

LOO7-HA5:ES1 protein was equilibrated with 10-fold molar excess of target HA5 peptide (conc. approx. 1 mM) in P buffer (10 mM sodium phosphate and 5 mM sodium chloride at pH 8.0) overnight and concentrated by diafiltration using a 3 kDa filter. The product was then run on a Superose 12 GL300 size exclusion column with a bed volume of 24 mL at a flow rate of 0.5 mL/min using P buffer as the mobile phase. The eluted protein was monitored by measuring the absorbance at 280 nm. Protein that was not equilibrated with the target peptide was run using the same procedure, for comparison. Molecular weights for elution peaks were estimated by retention times, comparing to the *Gel Filtration Standard* (Bio-Rad, Hercules, CA, USA).

### 2.6. LOO7-HA5:ES1 Peptide Binding Affinity

Fluorescence-based manual mixing experiments were used to measure the binding kinetics of LOO7-HA5:ES1 for the HA5 peptide. Synthetic and >95% pure peptide (GenScript, Piscataway, NJ, USA) was used for all binding experiments. First, 300 μL of peptide at varying concentrations was mixed with 2700 μL of 0.1 μM LOO-GFP biosensor in P buffer. The peptide concentrations used were all super-stoichiometric, from 0.1 μM to 100 μM. The upper and lower limits for signal amplitudes were determined by starting with 100 μM peptide and lowering the concentration in a 2-fold dilution series. The upper limit of peptide concentration is defined here as the concentration of peptide at which the signal amplitude was maximum. The lower limit was obtained by dilution with 300 μL buffer. Fluorescence trajectories were recorded for 300 s with a time step of 0.1 sec on a Fluorolog Tau 3A spectrofluorometer (Jobin-Yvon Horiba, Irvine, CA, USA), in a 3.5 mL quartz cuvette (Starna, Essex, UK). Excitation and emission wavelengths were set to 472 nm and 499 nm, with 3 nm and 5 nm slit widths, respectively.

Fitting the fluorescent traces was complicated by the rapid light-induced first-order decay. To factor out this decay, the fluorescence trace was fit as follows. (1) Counts were normalized to the starting value (4.0 ± 0.5 × 10^5^ counts/s) for each run. (2) Light-induced first-order fluorescence decay was fit to the times before (k = 0.002 s^−1^) and after (k = 0.001 s^−1^) addition of the peptide. (3) The difference between these two baselines, after accounting for the dilution factor (0.9 or 0.95), was taken as the peptide-induced fluorescence gain. The rate of peptide-induced fluorescence gain was fit (k_b_ = 0.37 s^−1^ ± 25%) by global, non-linear least squares. Fits were validated by a flat, zero residual.

### 2.7. Fluorescence Quantum Yield

For all variants discussed in this paper, the relative fluorescence quantum yield (QY) was calculated by measuring counts-per-second (cps) at 508 nm with 485 nm excitation on a Fluorolog Tau 3A spectrofluorometer using a 3.5 mL quartz cuvette with 1 cm pathlength, using slit widths 2 nm and 2 nm for excitation and emission, respectively. Measured cps was compared to that of 1.0 μM sfGFP-OPT in P buffer at pH 8 measured using the same settings. The ratio was scaled by the μM concentration of the variant to obtain the QY.

### 2.8. SGMU Fiber Preparation

Cloning, purification, and fiber formation for fusion proteins eGFP-Ubx, mCherry-Ubx, and LOO8-GFP-Ubx are described elsewhere [15]. The new construct presented here is a multipart fusion composed of Sortase A (SrtA), LOO-GFP, maltose-binding protein (MBP), and ultrabithorax (Ubx). The construct SrtA-LOO11-GFP-MBP-Ubx is named as “LOO11-SGMU” (see Supporting Data). The construct includes an N-terminal histidine tag followed by the target (priming) peptide, followed by SrtA, followed by the SrtA cut site, followed by LOO-GFP, then MBP, then Ubx.

E coli BL21(DE3) cells expressing SGMU constructs in the pET28a+ vector were induced with 1 mM IPTG and allowed to grow overnight at 28 °C. The cell pellets were resuspended in binding buffer (50 mM Na_2_HPO_4_, 500 mM NaCl, pH 8) with *Protease Inhibitor Cocktail* and DNase (Thermo Fisher Scientific, Waltham, MA, USA) added. Cells were lysed by sonication. Cell debris (pelleted for 30 min at 12,000 rpm) was discarded, and the supernatant was filter sterilized using a 0.45 mm filter. LOO11-SGMU protein was purified from lysate using a *HisTrap* 5 mL affinity column washed with wash buffer (50 mM Na_2_HPO_4_, 500 mM NaCl, 100 mM imidazole, pH 8) and eluted with elution buffer (50 mM Na_2_HPO_4_, 500 mM NaCl, 500 mM imidazole, pH 8). SDS-PAGE/Western blotting showed the expected 130 kD band, along with several degradation products.

A shallow metal tray was filled to the brim with fiber buffer (50 mM NaH_2_PO_4_, 500 mM NaCl, 5% glucose, pH 8) with the buffer domed slightly above the edges of the tray. Then, 2–3 mL of purified SGMU at a concentration of 3 mg/mL was layered over the buffer, drop by drop, moving the pipette around the tray while dropping and without stirring. The tray was protected overnight with an aluminum foil tent, not touching the tray or the liquid. Tray was left at room temperature from 6 h to 2 days, typically 18–20 h. A plastic block (clean, 80-well microcentrifuge tube rack) was gently placed along the far rim of the tray. Buffer was gently added below the surface using a serological pipette, so as not to disrupt any biomaterial that had formed on the surface, as needed to make the buffer surface contact with the plastic block. The block was pulled slowly across the tray until 2–5 cm remained. This procedure collected all the floating biofilm into a smaller area. By placing the tip of a paper clip just below the surface and slowly pulling, a fiber formed, which was wrapped around the paperclip. All experiments with LOO11-SGMU were carried out by soaking the fibers on the paper clip in various solutions and imaging in an inverted fluorescence microscope using excitation wavelength of 450–490 nm and emission wavelength of 520–560 nm, using band-pass filters.

*SGMU Experiment 1.* A fiber was imaged. The fiber was washed with pH 2 10 mM GlycineHCl buffer for 10 min (fluorescence disappears). The fiber was washed with pH 8 50 mM sodium phosphate buffer for 10 min.

*SGMU Experiment 2.* A fiber was imaged. The fiber was soaked in 100 mM CaCl_2_ for 10 min. Experiment 1 follows. Fluorescence is not expected to reappear.

*SGMU Experiment 3.* Experiment 2 is carried out. The fiber was washed with 1 mM target peptide in pH 8 50 mM sodium phosphate buffer for 10 min, then reimaged. Alternatively, fiber was soaked in a solution containing a heat-denatured protein containing the target sequence.

## 3. Results

### 3.1. Library Screens

**Figure 2:**
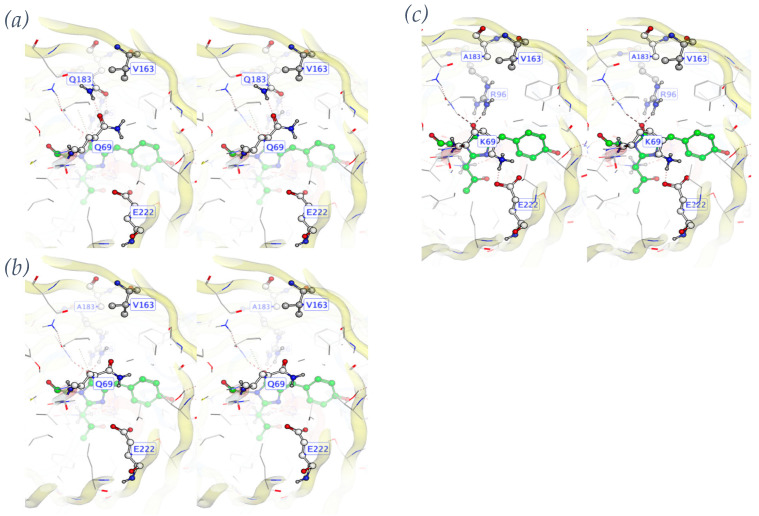
Domino effect in the LOO8-NS1#2 library. (**a**) Target mutation A163V creates clash with Q183. (**b**) Q183A alleviates clash but leaves Q69 unsatisfied, compromising protein folding. (**c**) Q69K makes salt bridge with E222. CRO loses catalytic E222 positioning, preventing chromophore (green atoms) formation.

A full and detailed analysis of each of the eight computationally designed and experimentally screened libraries is provided as Supplementary Data. In summary, we found that there were two issues with the Rosetta force field that explained the results. Close packing of side chains is rewarded instead of penalized. Also, unsatisfied hydrogen bonds are not penalized enough. Along with these issues, we also observed GFP-specific challenges in protein design, namely a rugged fitness landscape. An example of ruggedness in a fitness landscape is given in Figure 2, which shows a “domino effect” of design choices. Ruggedness means that there is no simple mutational pathway from one folded and functional state to another. Instead, mutations are interdependent. A sequence may be unfit in several ways. It may not fold, or not mature the chromophore, or not bind the peptide, or it could dimerize in the unbound state. We hypothesize that the rugged landscape arises from the beta barrel topology, which is like a closed container with no room to expand or contract in the core.

### 3.2. LOO7-HA5:ES1 Peptide Binding

**Figure 3:**
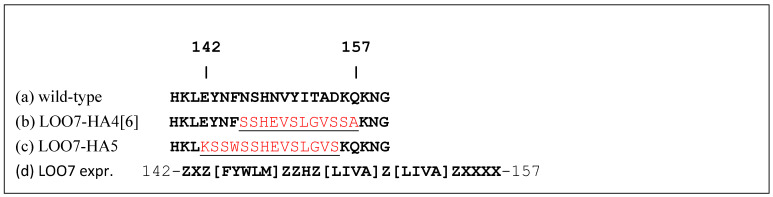
Left-out strand and targets. (**a**) Wild-type sequence of strand 7 (residues 139–160) from sfGFP-OPT [8]. (**b**) LOO7-HA4 strand 7 sequence with target peptide HA4 (**red**) derived from residues 139–150 of Influenza A virus (A/Thailand/16/2004(H5N1)) hemagglutinin. (**c**) LOO7-HA5 strand 7 sequence with target peptide HA5 (**red**) derived from residues 135–148 of Influenza A virus. (**d**) PROSITE expression [28] used to screen for candidate LOO7 target sequences, where X={C},Z={CP}.

**Figure 4:**
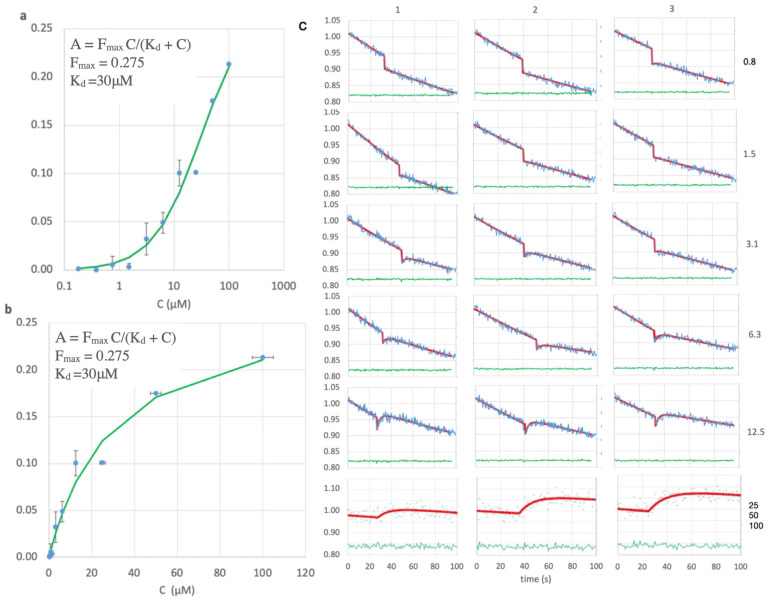
Peptide binding. (**a**) Log and (**b**) linear scale plots for peptide concentration, C, versus observed signal amplitude, showing fit (green) to the Langmuir equation to give K_D_ = 30 μM (r = 0.99). (**c**) Fluorescence (blue, ex485/em508) versus time after opening the shutter for all peptide concentrations (right side, μM), in triplicate (top margin) except for 25–100 μM. The Y-axis for each in relative counts (blue lines, points) normalized to the starting value for each run. Concentrations 25–100 μM were run at a 2× higher dilution and smaller slit width. Red: least squares fit. Green: residual of the fit.

**Figure 5:**
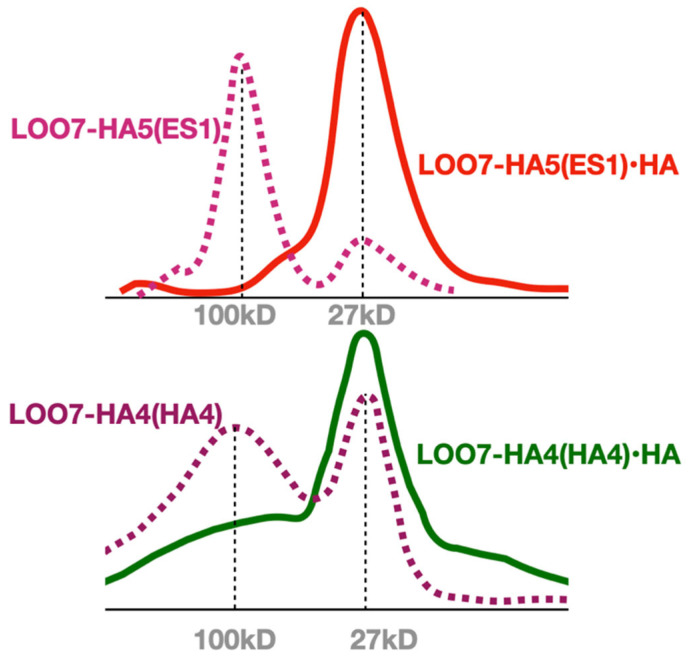
Multimer to monomer transition. Size exclusion chromatography of HA-peptide-bound and unbound LOO7-HA constructs. Dominant multimeric state is apparently tetramer.

Colony ES1 (see Supplementary Data), the only positive in the LOO-format LOO7-HA5 library screen, underwent further study. LOO7-HA5:ES1 was expressed and purified as described above, and peptide-induced fluorescence kinetics was measured. After correcting for light-induced fluorescence decay, amplitudes of peptide-induced fluorescence gain were accurately measured and fit to peptide concentrations (r = 0.99) using the Langmuir equation for single-site isothermal binding [43]. The result K_d_ = 30 µM is slightly weaker than the K_d_ = 9.0 µM affinity previously reported for LOO7-HA4:DS2 using the shorter HA4 peptide [4] (Figure 3). The kinetics of peptide-induced fluorescence gain (Figure 4) was fit to a first-order inverse exponential decay equation, giving rate k_b_ = 0.4 s^−1^ (t_1/2_ = 1.7 s) which is consistent with protein folding being the rate-limiting step [4]. SEC was performed with and without the HA5 peptide, showing a transition from a multimeric state to a monomeric state when peptide is added (Figure 5). Similar SEC results were seen for LOO7-HA4:DS2 [4] and for LOO7-GFP [3]. The major difference is that LOO7-HA4:DS2 rapidly lost fluorescence upon addition of the HA4 peptide, while LOO7-HA5:ES1 gains about 27% in QY relative to the unbound, multimeric state, when the peptide HA5 is added.

**Figure 6:**
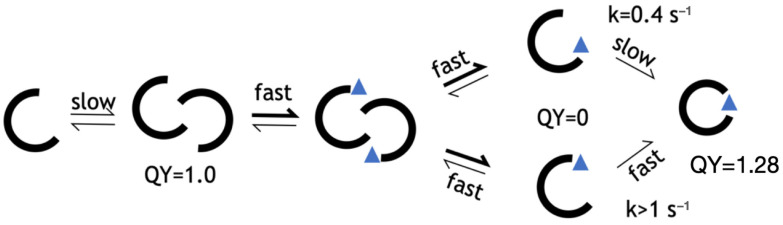
Kinetic model for HA5. From left to right: unbound monomer, fluorescent unbound multimer, peptide-bound multimer, peptide-bound monomer with open barrel, fluorescent peptide-bound monomer with closed barrel. Values for QY and rate determined in this work.

Having shown that folding is the rate-limiting step for a peptide-induced 27% increase in the QY, we can assert that the final bound state of LOO7-HA5:ES1 is a ***closed barrel***, while that of LOO7-HA4:DS2 (whose rate of fluorescence signal change was much too fast to be due to folding) is an ***open barrel***. Our working model for the LOO-GFP system (Figure 6) includes a dimeric, open-barrel, unbound state and a monomeric, closed-barrel, bound state. The lack of barrel closing is attributed to overpacking, specifically at W83 in LOO7-HA4:DS2. In energy-minimized and rotamer-optimized models, W83 makes close contacts with N185 on one side and L195 on another side. On the other hand, F83 in LOO7-HA5:ES1 adopts the same rotamer but has no such clashes. A tighter interaction implies a higher entropic barrier to barrel closing.

### 3.3. LOO7-HA5:ES1 Photo-Switching and Blue Shift

Peptide-bound LOO7-HA5:ES1•HA5 shows a first-order decay in fluorescence upon excitation with 471 nm light. One of the other fluorescent members of the LOO7-HA4 library, a clone called LOO7-HA4:DS2 [6], also carried an AYG chromophore (heterogroup PIA in 5DTZ [44]) showing a similar albeit less pronounced light-induced first-order decay. This decay is due to the intrinsic properties of the PIA chromophore, which is known to confer reversible photo-switching ability [44]. Upon irradiation, the chromophore switches to the *trans* stereoisomer, quenching fluorescence. In some cases the stereoisomer switches back spontaneously and 471/499 excitation/emission returns. In other cases, the reversal back to *cis* requires irradiation at 405 nm [45]. Since we were not interested in photo-switching, we made the reversion mutant LOO7-HA5:ES1/A65T to recover the wild-type TYG chromophore (heterogroup CRO), however, this resulted in no fluorescence. The appearance of colonies of the A65T revertant lacked the tint we have come to associate with a mature chromophore, therefore we speculate that the A65T revertant mutation blocked chromophore maturation in the context of the other mutations in the complex LOO7-HA5:ES1/A65T•HA5.

**Figure 7:**
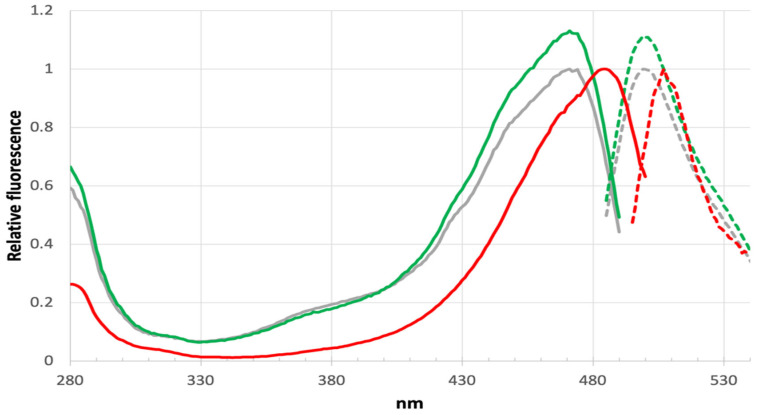
Excitation/emission spectra.Excitation (solid lines) and emission (dashed lines) spectra for unbound LOO7-HA4 (red), unbound LOO7-HA5:ES1 (grey), and bound LOO7-HA5:ES1•HA5 (green). For LOO7-HA5:ES1, excitation peak is at 471 nm, emission peak is at 499 nm.

A significant blue shift in excitation and emission is found for LOO7-HA5:ES1•HA5 versus LOO7-HA4:DS2•HA4 or wild-type GFP, with peak excitation at 471 nm and peak emission at 499 nm, shifted by −13 and −9 nm, respectively (Figure 7). T65A removes a hydrogen bond donor from to the N2 imidazolidone ring nitrogen, destabilizing the anionic form of the PIA chromophore. By removing that stabilizing interaction, the PIA chromophore exists more in the neutral form and its less extensive conjugation absorbs higher-energy photons. In fact, comparison of the excitation/emission spectra with wild-type GFP shows not only the blue shift but also a significant increase in excitation at lower wavelengths (350–400 nm). The neutral form of the chromophore absorbs at the lower end of the 350–490 nm excitation range [46]. The slightly increased Stokes shift in LOO7-HA5:ES1•HA5 could be due to the loss of T65 OG, which is one of the atoms in the excited state proton transfer pathway [47,48].

### 3.4. Experiments on Fibers

The previous fiber-based LOO-GFP construct [15], called LOO8-GFP-Ubx, enhanced peptide-induced fluorescence, and eliminated the background fluorescence from dimerization. Therefore, deploying LOO-GFP biosensors on Ubx fibers may be a strategy to improve biosensor function. But it revealed an electrostatic interference problem between LOO-GFP and the highly charged fiber-forming protein Ubx. Quantum mechanical simulations suggest that GFP quantum yield is very sensitive to the electrostatic environment [49]. We sought to improve the deficiencies with the Ubx fiber system noted in our pilot studies. First, in the new SGMU construct we have inserted a 43 kD spacer, MBP, between LOO-GFP (27 kD) and Ubx (40 kD). This insertion should reduce intramolecular LOO-GFP:Ubx interactions in the fiber. Second, not all of the LOO8-GFP-Ubx monomers incorporated the co-expressed target peptide, therefore we included the target peptide in the same chain, separated by (and removable by) a calcium-activated self-cleaving protease, SrtA. Third, we used LOO11 instead of LOO8 to avoid a patch of negatively charged side chains near strand 8 that we hypothesized allowed LOO8:Ubx ionic interactions to block peptide binding.

**Figure 8:**
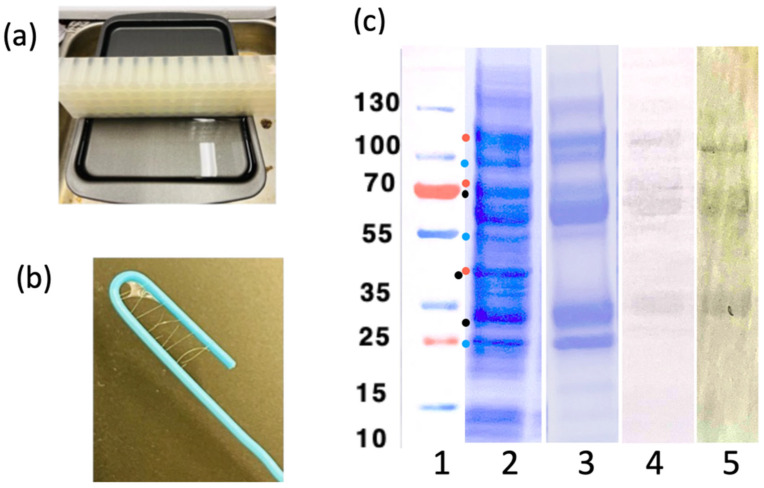
Biosensor fibers. (**a**) Buffer reservoir setup, used for making the fibers. (**b**) LOO11-SGMU fibers, approx. 100 μm thick, wrapped around a paper clip. (**c**) SDS-PAGE analysis of the LOO11-SGMU lysate before/after HisTrap (Ni-NTA) column chromatography. (Lane 1) Precision plus protein standards marked in kilodaltons. (Lane 2) Lysate. (Lane 3) HisTrap elution. (Lane 4) Western blot of elution using anti-Ubx. (Lane 5) Western blot of elution using anti-Histag. All gel images are aligned using standards. Dots in lane 1 are possible degradation product sizes based on locations of linkers. Blue dots contain the N-terminal Histag. Red dots contain the C-terminal Ubx. Black dots have neither.

Figure 8 shows the purification and the fiber-making setup. LOO11-SGMU is estimated to be at least 90% pure. The extra bands are degradation products since they react with either an anti-His tag antibody or an anti-Ubx antibody. Floppy linkers holding the component domains together are likely to the be sites of protease cleavage. It is possible that proteolytic cleavage is off-target activity of SrtA, a component of SGMU. Fortunately, the amount of full-length protein in the mix was sufficient to make fibers and characterize them. Ubx self-purifies as it forms fibers [50].

To test whether the altered SGMU construct could unfold and refold in fibers, we denatured and renatured as described in SGMU Experiment 1. Unfolding and refolding LOO11-SGMU recovered approximately 50% of the original fluorescence in the presence of the covalently linked target peptide. The incomplete recovery was expected, due to the well-known “trapped state” of refolded GFP, in which approximately half of the chromophore takes on the non-fluorescent trans state [51]. Encouragingly, the partial recovery of fluorescence on refolding required no added salt or detergent, in contrast to the previous construct, LOO8-GFP-Ubx, which needed high salt to prevent irreversible electrostatic interference from Ubx. We conclude that inserting MBP between LOO-GFP and Ubx diminished the electrostatic effect.

**Figure 9:**
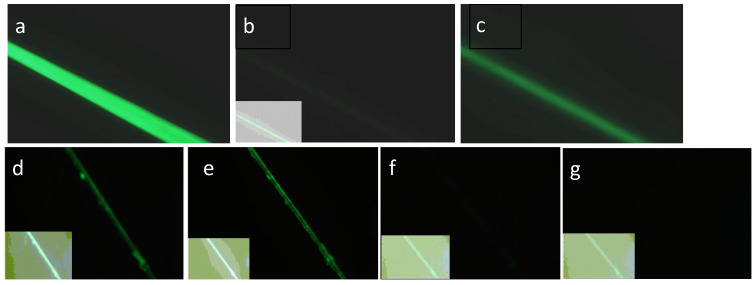
Results of SGMU Experiments 1, 2. (**a**) LOO11-SGMU fiber in fluorescence microscope using Nikon Blue filter. (**b**) Same fiber after glycine pH 2 buffer added. Inset in each case is an enhanced image optimized for contrast, showing presence and location of fiber. (**c**) Same fiber after phosphate pH 8 buffer added, observed for 5 min. Here, 35% of the original fluorescence returned. (**d**) Different LOO11-SGMU fiber under microscope using Nikon Blue filter. (**e**) Same fiber after CaCI_2_ buffer added, observed for 10 min with 40% loss in brightness. (**f**) Same fiber after glycine pH 2 buffer added, observed for 5 min. Less than 1% of brightness remains. (**g**) Same fiber after PBS pH 8 buffer added, observed for 5 min. No fluorescence is recovered. Signal-to-noise ratio is panel (**c**) divided by panel (**g**).

SGMU Experiment 2 produced complete loss of fluorescence after the target peptide was removed by activating the SrtA with CaCl_2_ (Figure 9). This shows that the unbound state of LOO11-GFP is not forming dimers on the fiber as is does in solution and that only the peptide-bound state is fluorescent. This is remarkable because the fibers were composed of 100% LOO11-SGMU, whereas in the pilot study the concentration of the sensor fusion had to be reduced to 10% (10% LOO8-Ubx, 90% plain Ubx) in order to remove the background fluorescence [15].

In SGMU Experiment 3, the addition of exogenous peptide did not deliver the expected fluorescence recovery. A saturating amount of s11 peptide was added to the fiber for 10 min, but no appreciable increase in fluorescence was observed. We also added an unfolded GFP experimental variant (itself non-fluorescent) containing the s11 sequence, but this too did not reconstitute fluorescence. This suggests that there may still be electrostatic interference, despite the MBP spacer. Therefore, the SGMU design solves several of the issues encountered in the pilot study. Work is ongoing to solve this electrostatic problem.

## 4. Discussion

### 4.1. Biosensor Library Screening

Creating LOO-GFP biosensors by computational design and plate screening for fluorescence encountered several obstacles, and each obstacle led to a solution or at least a partial solution, and each came with a lesson. First, we found that libraries in which many residues are designed failed to produce a functional protein sequence whether the designable positions were sampled at high complexity (many sequence permutations) or at low complexity. High complexity screening relies more on the number of colonies screened, while low complexity depends more on the computational design accuracy. The beta barrel structure of GFP is unusually stable [52] and has been described as having a “badlands” sequence fitness landscape [27], characterized by steep energy differences and highly covariant energetic effects of mutation. We describe one such covariant or “domino effect” in Figure 2. Sampling a badlands energy landscape is difficult because optimal residue choices depend on other residue choices, in a chain of dependency. The search space paradox was resolved by focusing on a smaller design space, using the piecemeal approach [16]. Functional, fluorescent sequences were found when the number of designed positions was 15 or fewer, whether the library complexity was high, low, or medium. This sweet-spot design space size corresponds to the nearest neighbor residues of four to five consecutive positions of the target peptide. Thus, a full-length biosensor design can be accomplished in three “pieces”, using the piecemeal design approach [16].

But underlying the search space problem were at least two defects in the energy function of design. Too often side chains were packed together tightly instead of finding loosely packed solutions. Loose side chain packing provides less of an entropic barrier to folding. In the spirit of flexible packing, we experimented with a design protocol called plastic protein design (PPD) [32], used with Rosetta. This method favors amino acid choices that are compatible with backbone motions. But PPD did not prevent overpacking, and several examples were found where overpacking was the most likely cause of the failure of folding, binding, or catalyzed chromophore maturation. We hereby assert that *an energy function for design should reward loose packing instead of rewarding close contacts*. The entropy gained should more than compensate for the loss of the short-ranged van der Waals attractive term. Alternatively, the attractive vdW force could be extended to a carbon–carbon distance of 5.8 Å to capture the attractive force of desolvation [53]. Another shortcoming of the Rosetta scoring function, until recently, was its lack of a penalty on hydrogen-bonding groups that are unsatisfied and buried in the core [54].

The three-step screening process (native, LII, LOO) was intended to gradually increase the selection pressure. Our strategy was to first express all combinations of all mutations in the native format, then pick fluorescent individuals and sequence them. Second, we would express the selected sequences as a circular permutant with the target sequence at the new N- or C-terminus, then pick fluorescent individuals again and sequence them. Finally, we would screen the selected mutations (all combinations) as LOO-GFPs with the target expressed exogenously as a fusion to a separate protein. It was believed that many mutations would be tolerated in the native format but that circular permutation would impose an additional energetic penalty, allowing the selection to identify the most stable designs. The LOO format imposes an additional energetic penalty by making it two pieces, intensifying the selection pressure. Though fluorescent colonies were obtained up to the LII stage, none of the selected individuals shortlisted at this stage were fluorescent as LOO-GFPs. These results and those published previously [17,18,19,25] suggest a rough fitness landscapes of intact GFPs and their self-assembling split counterparts.

### 4.2. LOO-GFP Biosensors on Fibers

Because the stability of the LOO-GFP variants appears to limit the successful identification of active variants in a library, a universal approach to stabilize these proteins was needed. Proteins genetically fused to Ubx and incorporated into materials are remarkably stabilized. For instance, enhanced GFP (EGFP) in Ubx materials can withstand boiling, submersion in organic solvents, and even autoclaving conditions [55]. Thus, embedding LOO-GFPs in Ubx materials may impart function in variants incapable of folding into monomers. This approach has the added benefit of immobilizing the LOO-GFP, facilitating analysis.

LOO8-GFP was added N-terminally to Ubx, and the purified construct fluoresced and formed fibers as described [15]. In that work, the priming strand could be removed by dropping the pH to 2. Binding of exogenous s8 peptide was demonstrated and coincided with a gain of fluorescence, culminating in the proof of concept of a biosensor screening system with almost zero background fluorescence and almost no possibility of dimerization or aggregation. But this success was achieved only after solving a mysterious and seemingly permanent loss of function after unfolding and refolding. The loss was attributed to electrostatic interactions between LOO-GFP and Ubx, because adding high salt (1.25 M NaCl) prevented the loss of biosensing [15].

Another solution to the electrostatic interference problem was to introduce MBP in between LOO-GFP and Ubx. The construct s11-SrtA-LOO11-GFP-MBP-Ubx (see Supplementary Data) formed fibers, fluoresced, and was able to recover fluorescence (about 50%) after unfolding and refolding using pH shift in the presence of the covalently linked target peptide (s11). No salt or detergent was required in this case. There was no fluorescence if the target peptide was removed by activating the sortase A (SrtA) followed by washing, solving the fluorescent multimer problem. The recovery of 50% of the fluorescence was expected since even full-length, wild-type GFP recovers only about half of the fluorescence upon unfolding and refolding [51].

### 4.3. Influenza Biosensor Design

In this work we re-engineered an existing LOO7-GFP biosensor for H5N1 hemagglutinin. The key objectives were to obtain a gain of fluorescence upon binding and a higher specificity, using the wild-type strand 7 peptide as the control. While both goals were met, the current prototype still binds to the wild-type strand 7. Even the dramatic change caused by Y173S was not sufficient to find a design that excluded the original peptide from binding it. This points to an inherent conflict in this type of design problem. We want to make the fewest and most conservative changes to be assured of finding a successful sequence, but on the other hand we want to include dramatic changes so that binding specificity is maximally shifted to the new target. That goal remains.

## 5. Conclusions

The LOO-GFP format provides a promising, self-reporting, protein-only biosensor system that can be computationally designed and expressed in bacteria. It can even be immobilized on fibers to make biosensor materials. The challenges encountered in creating this capability were both computational and GFP-specific. A LOO-GFP biosensor needs to recognize and bind at least a 12-residue peptide, requiring changes at an estimated 28 to 38 positions. This creates a double-bind, a high enough library complexity is both necessary for success and out of reach for experimental screening. We solved this problem by dividing the design space into manageable pieces, a method called “*piecemeal*”. Three rounds of *piecemeal* design are needed to design a biosensor. Underlying the library complexity problem was the ruggedness of the fitness landscape. Observed domino effects in design choices means that functional sequences are sparce, with highly interdependent mutations. Underlying the energy landscape is the energy function, in which we discovered two critical defects. The first is the rewarding of overpacking of side chains in the core. An energy function for design should reward loose packing instead of tight packing. The second is the absence of a penalty for missing hydrogen bonds. Also, the LOO-GFP method itself has an inherent weakness in that the unbound state is an open barrel that can form soluble homomultimers. Our past efforts, including the ones described in this work, were constrained to solve two often conflicting optimization problems of (a) increasing binding affinity and (b) preventing homomultimerization. Therefore, most importantly, we show that immobilization of LOO-GFP on Ubx fibers simplifies the optimization to only one goal, which is increased binding affinity. It also vastly improves the signal-to-noise ratio, providing higher dynamic range and sensitivity for detection of pathogens at low titers. We envision a microliter-sized fluorometer flow cell packed with LOO-SGMU fabric, for use in a lab setting or in automated mosquito traps to monitor for the presence of mosquito-borne viruses.

## Figures and Tables

**Table 1:** Sequence libraries.

Left Out	wt	Gene Library Sequence Expression Target [Designed] Hits CRO Triplet in Bold, / / N,C Termini	Designed (Variable), Complexity
Name	Target Piece		Glowing / Total (Format)
sfGFP-OPT		SMASKGEELFTGVVPILVELDGDVNGHKFSVRGEGEGDATIGKLTLKFICTT GKLPVPWPTLVTTL**TYG**VQCFSRYPDHMKRHDFFKSAMPEGYVQERTISFKD DGKYKTRAVVKFEGDTLVNRIELKGTDFKEDGNILGHKLEYNFNSHNVYITA DKQKNGIKANFTVRHNVEDGSVQLADHYQQNTPIGDGPVLLPDNHYLSTQTV LSKDPNEKRDHMVLLEFVTAAGITHGMDELYK	
LOO8	NGIKANFKIRHNV	/MA SKGEELFTGVVPILVELDGDVNGHKFSVRGEGEGDAT[IN]GKL T LKFICTTGKLPVPWP[ST]LV[AT]TL**TYG** VQCFSRYPDHMKRHD F [AF]KSAMPEGYVQERTISF[KQ]DDG[KT]YKTRA[EV]VKFEG D TLVNRIELKG[IT]DFKEDGNILGH[KQ][LV][ER]Y[NS]F[NS] [ES]H[MN][TV][KY][IL][ST]A[DS][KQ][NQ][KS] sgifitdnvhtwt [EL][DN]GS[IV]Q[LV]A[DS][HV][DY][QS][AQ] [NK][TY]PIGDGPVLLPDNHYLS[TV]Q[ST]VLSKDPNEKRDHMV LLEFVTAAGITHG/	36(36), 6 × 10^10^
NS1#1	** SGIFITDNVHTWT **		**0**/1000 (native)
LOO8	NGIKANFKIRHN V	/MA SKGEELFTGVVPILVELDGDVNGHKFSVRGEGEGD AT[IN] GKLT LKFICTTG KLPVPWP[ST]LV[AT]TL**TYG**V[KQ]CFSRYP DHMKRHD[FY] [FL]KSAMPEGYVQERTISF[KQ]DDG [KT]YKT RA[EV]VKFEGDTLVNR IELKG[IT]DFKEDGNILGH[KR] [LV][ER]Y[NS] [FS][NS][ES]H[HN][EV][KY][AI]T [AM]D[GK][NQ][EK]ygfgvfttniwlkE [DG]GS[VY][QR] [LM]A[DH][DH][KY][AQ][FQ]N[ST]PIGDGPVLLPDN[HY] YLS[GT]Q[ST]V LSKDPNEKRDHMVLLEFVTAAGITHG/	38(38), 1.4 × 10^11^
NS1#2	** YGFGVFTTNIWL ** ** K **		**0/**1000 (native)
LOO11	DHMVLLEFVTAA	/MASKGEELFTGVVPILVELDGDVNGHKFSVRGEGEGDAT[N]G[KQ] [IL][I]L[R][IL][V][N]TTGKLPVPWPTL[A]TTL**TYG**VQC Y[FINY][K][T][T]DH[KQ]KRHDFFKSAMPEGYVQERTISF KDDGKYKTRAVVKFEGDTLVNRIELKGTDFKEDGNILGHKLEYN [I][N]SHNVYITADKQKNGIKANFTVRHNVEDGSVQLADHYQQNTPIGDG PVLLPD[Y]H[N][S][Q]T[N][ST][Y][FY][ST]K[IL][T]NEKR dmgywiesalndGITHGMDELYK/	37(9), 2048
DMG	** DMGYWIESALND **		**0**/1000 (native)
LOO8	NGIKANFTVRHN V	/MASKGEELFTGVVPILVELDGDVNGHKFSVRGEGEGDATIGKLTLKFICTT GKLPVPWPTLV[S]TL**TYG**VQCFSRYPDHMKRHD[Y][Y]KSAMPEGYVQER TIS[T]KDDGKYKTRAVVKFEGDTLVNRIELKGTDFKEDG[I]ILGHK[I] [Q]YN[IL]QR[HPSY][R][IV][Y][H][W][Q]D[W][S][N] ngtkgdftngnst E[N]GSV[AV]F[HKNQ][DH][A][E]QQ[S][HKNQ][A]IGQHQVLL [A]DNHYLSTQTVLSKDPNEKRDHMVLLEFVTAAGITHGMDELYK/	28(7), 1024
HIV	** NGTKGDFTNGNST **		**0**/1000 (native)
LOO8	NGIKANFKIRHNV	/ MASKGEELFTGVVPILVELDGDVNGHKFSVRGEGEGDATIGKLTLKF ICTTG KLPVPWPTLVTTL**TYG** VQCFSRYPDHMKRHD[I][F]KSAMPEGYIQERT ISFKDDGKYKTRAVVKFEGDTLVNRIELKGTDFKEDGNILGHKLEYNFN SHNVYITA[ DE ][DEGHIKNMQRSV][GE][DEGHIKNMQRSV] yg IKAN FKIRHNVEDGSVQLADHYQQN[ AST]PIGDGPVL[LV]PDNHYLSTQTV LSKDPNEKRDHMVLLEFVTAAGITHG/	9(6), 3432
NS1#3	** YG ** FGVFTTNIWLK		**10**/65 (native)
LOO8	NGIKANFKIRHNV	MASKGEELFTGVVPILVELDGDVNGHKFSVRGEGEGDATIGKLTLK FICTTG KLPVPWPTLVTTL**TYG** VQCFSRYPDHMKRHD[ I ][F ]KSAMPEGYIQERT ISFKDDGKYKTRAVVKFEGDTLVNRIELKGTDFKEDGNILGHKLEYNFN SHNVYITA[ DE][DEHKNQRS][GE][DEH KNQRS] // yg IKANFKIRHNVED GSVQLADHYQQN[AS T]PIGDGPVL[LV]PDNHYLSTQTVLSKDPNEKRDH MVLLEFVTAAGITHGMDELYKGGTGGS	9(6), 1536
NS1#4	** YG ** FGVFTTNIWLK		**5**/70 (LII)
LOO7	FNSHNVYIT	MASKGEELFTGVVPILVELDGDVNGHKFSVRGEGEGDATIGKLTL KFICTT GKLPVPWPTL[DENKQRSTV][NST]TL**TYG**VQCFSRYPDHMKRHD[AF LM] FKSAMPEGYVQERTISFKDDGKYKTRAVVKFEGDTLVNRIELKGTDFKEDG NILGHKLEYN /nsvtniele/ ADKQKNG [AGILV]K[AG][DEKNQR] F[HQ RTNK] [KQT NS]RHNVEDGSVQLADHYQQNTPIGDGPVLLPD[K NQR] H[KNQR]LST[DE NKQRY ][N ST ]V[ALVNQ]SKDPNEKRDHMVLLEFVTAA GITHGMDELYKGGTGGS	13(13), 3.9 × 10^9^
EDIII	** NSVT ** NIELE NSVT**NIELE**		**0,2**/250 (native), >**4**/250 (LII), **0**/250 (LOO)
LOO7	NSHNVYITAD	MSKGEELFTGVVPILVELDGDVNGHKFSVRGEGEGDATIGKLTLKFI CTTGKLPVPWPTLVTTL**TYG**VQCFSRYPDHMKR HD[FW][ F M]KSA MPEGYVQERTISFKDDGKYKTRAVVKFEGDTLVNRIELKGTDFKEDG NILGHKLEYNF /sshevslgvs/ KQKNG[ I LV] K[ I ][NS T ][ F ] [T][IM V ][NS T ]HNVEDGSVQLADHYQQNTPIGDGPVLLPDNH [ H KR][FM L ][H K R][T][NS T ][T]VLSKDPNEKRDHMVLLEF [I V ]TAAGITHGMDELYKGGT GGS	16(11), 34992
HA4	** SSHEVSLGVS **		**75**/2500 (LOO)
LOO7	EYNFNSHNVYITAD	MASKGEELFTGVVPILVELDGDVNGHKFSVRGEGEGDAT[IN]GKLTL KFICTTG KLPVPWPTLV[AGT]TL**[AT]YG** VQCFSRYPDHMKRHD [CFLW][F]KS[AT ] MPEGYVQERTISFKDDGTYKTRAEVRFEGDTLVNRIELKGIDFKEDGNILGHKL /ksswsshevslgvs/ KQKNGIK [AGI][T]FT[AGV]R[HYFLV][DEK N] VEDGSVQLADHYQQNTPIG DGPVLLPDNH[HNSTY]L[K]T[T][AHYGST] VLS[KY]DPNEKRDHMVLLEFVTAAGITHGMDELYKGGTGGS	15(11), 276480
**HA5**	** KSSWSSHEVSLGVS **		**8**/50 (native), **1**/8 (LOO)

## Data Availability

Sequences and structures referenced in this work are available from NCBI using the accession codes in Appendix A or found in Supplementary Data.

## Supplementary Materials

The following supporting information can be downloaded at: https://www.mdpi.com/article/10.3390/s24196380/s1, Figure S1. SGMU amino acid sequence.; Figure S2. Sequence of LOO7-HA5:ES1.; 1. Supplementary Methods 1.1 Estimation of PCR bias during library amplification; 1.2 Internal hydration analysis; 2. Supplementary Results and Discussion 2.1. LOO8-NS1#1 high complexity dengue biosensor library; 2.2. LOO8-NS1#2 high complexity dengue biosensor library; 2.3. LOO11-DMG full-length, low complexity, dengue biosensor library; 2.4. LOO8-HIV full-length, low complexity, HIV biosensor library; 2.5. LOO8-NS1#3 small piecemeal library; 2.6. LOO8-NS1#4 small piecemeal library; 2.7. LOO7-EDIII high complexity, large piecemeal, dengue biosensor library; 2.8. LOO7-HA5 recalculated library, medium complexity; 2.9. PCR amplification bias not found [56,57,58,59,60].

## Appendix A

### Appendix A.1. Accession Codes

- Sortase A. partial (Staphylococcus aureus). GenBank: WP_084935205.1, PDB: 1T2W.
- Maltose binding protein maltose/maltodextrin ABC transporter substrate-binding protein MalE (Escherichia coli). GenBank: EFB2378405.1. PDB: 7MQ7.
- Superfolder green fluorescent protein (sfGFP), precursor to sfGFP-OPT. GenBank: UFQ89826.1. PDB: 2B3P.
- Ultrabithorax (Ubx), isoform F (Drosophila melanogaster). NCBI Reference Sequence: NP_996219.1.
- Hemagglutinin (Influenza A virus (A/Thailand/2(SP-33)/2004[H5N1])). GenBank: AAS65618.1.
- Dengue virus polyprotein (dengue virus type 2), includes cleavage products NS1 (776..1127) and E-protein (281..775). GenBank: WPF60237.1.
- Envelope glycoprotein (human immunodeficiency virus 1). GenBank: AEQ75975.1.
- Ssp dnaE intein (Synechocystis sp.). UniProtKB/Swiss-Prot: P74750.2.

## Appendix B

### Appendix B.1. Abbreviations

LOO, LOO7, LOO8, LOO11, leave-one-out protein or leave-one-out library. Number indicates GFP strand left out; LII, leave-it-in protein or leave-it-in library. Circular permutant of GFP; Native (format), library generated in the normal sequential GFP order with one strand mutated to the target sequence; GFP, green fluorescent protein; PCR, polymerase chain reaction; HA, hemagglutinin antigen; DNA, deoxyribonucleic acid; Ubx, ultrabithorax; MBP, maltose-binding protein; SrtA, sortase A; epPCR, error-prone PCR; DS, DNA shuffling; DS2, DNA shuffling clone 2; ES1, epPCR + DS clone 1; HA4, HA5, HA biosensor libraries; QY, quantum yield or relative quantum yield; vdW, van der Waals; PPD, plastic protein design; BR, Backrub; CCI, Center for Computational Innovations; MOE, molecular operating environment; MEC, minimum energy configuration; SM, simulated melting; EDIII, dengue e-protein biosensor library; NS1, dengue non-structural protein 1 biosensor library; HIV, human immunodeficiency virus, envelope protein biosensor library; BL21, strain of E coli; cps, counts per second; DEE, dead-end elimination; Kd, dissociation constant; CRO, TYG chromophore; PIA, AYG chromophore; SGMU, fiber-based biosensor material consisting of SrtA, LOO-GFP, MBP, and Ubx; s7, s8, s11, GFP strand 7, 8, or 11; SEC, size exclusion chromatography.
