# Supplementary material for: Challenges and Solutions for Leave-One-Out Biosensor Design in the Context of a Rugged Fitness Landscape
Source: Sensors (Basel). 2024 Oct 1;24(19):6380. doi: 10.3390/s24196380 (PMC11478963; doi:10.3390/s24196380)
Supplement: Supplementary file 1 [file sensors-24-06380-s001.zip › sensors-3237854-supplementary.pdf]

Supporting Material for

Challenges and Solutions for Leave-One-Out Biosensor Design in the Context of a Rugged Fitness Landscape

Shounak Banerjee[4, 1], Keith Fraser[1], Donna Crone[1], Jinal C. Patel[1], Sarah Bondos[3], Christopher Bystroff[1,2]*

[1]Biological Sciences, [2]Computer Science, Rensselaer Polytechnic Institute, Troy NY 12180, Rensselaer Polytechnic Institute, Troy NY 12180

[3]Medical Physiology, Texas A&M University, College Station, TX 77843

[4]Los Alamos National Laboratory, Los Alamos, NM 87545

*To whom correspondence should be addressed (bystrc@rpi.edu)

Supplementary Figures

Figure S1. SGMU amino acid sequence. Sources: **(Yellow)** Sortase A (SrtA) WP_084935205.1, partial [Staphylococcus aureus]. PDBid 1T2W, Residues 28 - 172.[56] (Underlined) SrtA protease recognition site. **(Green)** LOO11-GFP. Strand 11 sequence precedes Srt A.[12] **(Blue)** Maltose binding protein maltose/maltodextrin ABC transporter substrate-binding protein MalE [Escherichia coli]EFB2378405.1 Residues 29 - 392. [57] **(Salmon)** 10Histidine tag. Additional 6His tag and thrombin protease recognition site courtesy of pET28a+. **(Red)** Ultrabithorax [58]

MGSSHHHHHH SSGLVPRGSH MAHHHHHHHH HHSSGGHDDD DKDHMVLLEF VTAAGGSHGE
LTGDDGKPQI PKDKSKVAGY IEIPDADIKE PVYPGPATPE QLNRGVSFAE ENESLDDQNI
SIAGHTFIDR PNYQFTNLKA AKKGSMVYFK VGNETRKYKM TSIRDVKPTD VGVLDEQKGK
DKQLTLITCD DYNEKTGVWE KRKIFVATEV KGGKSGTGDS GLPETGGDSG TGDHGASGSG
SSGGITHGMD ELYKGGTGGS MASKGEELFT GVVPILVELD GDVNGHKFSV RGEGEGDATI
GKLTLKFICT TGKLPVPWPT LVTTLTYGVQ CFSRYPDHMK RHDFFKSAMP EGYVQERTIS
FKDDGKYKTR AVVKFEGDTL VNRIELKGTD FKEDGNILGH KLEYNFNSHN VYITADKQKN
GIKANFTVRH NVSSGHEDGS VQLADHYQQN TPIGDGPVLL PDNHYLSTQT VLSKDPNEKR
GGSGGTEFNE EGKLVIWING DKGYNGLAEV GKKFEKDTGI KVTVEHPDKL EEKFPQVAAT
GDGPDIIFWA HDRFGGYAQS GLLAEITPDK AFQDKLYPFT WDAVRYNGKL IAYPIAVEAL
SLIYNKDLLP NPPKTWEEIP ALDKELKAKG KSALMFNLQE PYFTWPLIAA DGGYAFKYEN
GKYDIKDVGV DNAGAKAGLT FLVDLIKNKH MNADTDYSIA EAAFNKGETA MTINGPWAWS
NIDTSKVNYG VTVLPTFKGQ PSKPFVGVLS AGINAASPNK ELAKEFLENY LLTDEGLEAV
NKDKPLGAVA LKSYEEELAK DPRIAATMEN AQKGEIMPNI PQMSAFWYAV RTAVINAASG
RQTVDEALKD AQTNGIEKLS SGSHGSNSYF EQASGFYGHP HQATGMAMGS GGHHDQTASA
AAAAYRGFPL SLGMSPYANH HLQRTTQDSP YDASITAACN KIYGDGAGAY KQDCLNIKAD
AVNGYKDIWN TGGSNGGGGG GGGGGGGGAG GTGGAGNANG GNAANANGQN NPAGGMPVRP
SACTPDSRVG GYLDTSGGSP VSHRGGSAGG NVSVSGGNGN AGGVQSGVGV AGAGTAWNAN
CTISGAAAQT AAASSLHQAS NHTFYPWMAI AGKIRSDLTQ YGGISTDMGK RYSESLAGSL
LPDWLGTNGL RRRGRQTYTR YQTLELEKEF HTNHYLTRRR RIEMAHALCL TERQIKIWFQ
NRRMKLKKEI QAIKELNEQE KQAQAQKAAA AAAAAAVQG GHLDQGS

Figure S2. Sequence of LOO7-HA5:ES1**.**

MGHHHHHHSSGKQKNGIKATFTVRHKVEDGSVQLADHYQQNTPIGDGPVL

LPDNHYLKTTGVLSKDPNEKRDHMVLLEFVTAAGITHGMDELYKGGTGGS

MASKGEELFTGVVPILVELDGDVNGHKFSVRGEGEGDATNGKLTLKFICT

TGKLPVPWPTLVTTLAYGVQCFSRYPDHMKRHDFFKSTMPEGYVQERTIS

FKDDGTYKTRAEVRFEGDTLVNRIELKGIDFKEDGNILGHKL

1. Supplementary Methods

1.1 Estimation of PCR bias during library amplification

Non-egalitarian PCR amplification was proposed as one reason for the failure to find a successful design in a sequence library. To test this hypothesis, one out of twenty 60-mer oligonucleotides (Integrated DNA Technologies, USA) for assembly PCR was modified to contain one of three restriction sites, yielding three polymorphs, containing either a BamHI, EcoRI or an NdeI site. Equimolar amounts of the polymorphic oligonucleotides and the remaining 19 oligonucleotides were pooled into a 70 µl assembly PCR mix. Assembly PCR was performed at annealing temperatures of 72, 67 and 64°C. All the assembly product was used to template a 50 µl Phusion polymerase-based PCR amplification. 4µl of amplification product was run on a 1% agarose gel. Correctly sized bands were selected and extracted using a Wizard SV Gel-Extraction/PCR-Cleanup kit (Promega, USA). In separate reactions 500 ng of purified gel extracts were digested with five units of BamHI, EcoRI and NdeI, for 90 minutes at 37°C. The products of the restriction digest were run on a 1% agarose gel to quantify each cut site.

1.2 Internal hydration analysis

The program DOWSER[39], was used to estimate the positions of internal water molecules in the biosensor candidates. For models containing chromophores, chromophore atoms were

separately parameterized using the Automated Topology Builder[59]. These selected designs were then visually inspected for the presence of unsatisfied hydrogen bonds. In DEEdesign, water orientations were treated as rotamers, with one rotamer option being no water[4].

2. Supplementary Results and Discussion

2.1. LOO8-NS1#1 high complexity dengue biosensor library

The target for this library was SGIFITDNVHTWT, residues 793-805 from dengue virus serotype 2 polyprotein (GenBank: UQM71438.1), whose cleavage product is non-structural protein 1 (NS1), replacing strand 8 sequence 159-NGIKANFKIRHNV-171. Although this library had a high theoretical sequence complexity (6.0×10^{10}), many of the variable positions (the target peptide positions are not variable) were not in the core, therefore not likely to be covariant. Hence they are likely to contribute independently to the energy. Only 9 positions in the core were variable, each with two choices, giving a core sequence complexity of just 512. For safety, one of the two choices was always the wild-type residue; the other was derived from computational design. Nonetheless, LOO8-NS1#1 gave zero positives. One possible reason is that target peptide sequence introduces invariant mutation F165D, which places a charge in the core near the catalytic R96. However, Asp at position 165 is observed in GFP homologs and a F165D point mutant of sfGFP-OPT was previously shown to fold and fluoresce[25], leading us to believe the buried charge would not be a problem. A second possible reason is that the target introduces A163I, creating a crowding situation. A compensatory shortening mutation Q183S is offered in the library, but this would destabilize the intricate hydrogen bonding network that positions R96 to catalyze formation of the chromophore. The only other position that could accommodate A163I is I152[IL], but neither choice at that position reduces crowding. DEEdesign erroneously rewarded crowding

in the HA4 design[4], and seems to be guilty of doing it again in the case of A163I. The only mutation that alleviates overcrowding is Q183S, but this breaks important hydrogen bonding. DEEdesign uses the Dead-end-elimination (DEE) algorithm to determine the global lowest free energy side chain configuration, but DEE locks sidechains into rotamers. Shrinking of the van der Waals radii by 0.9-fold in the energy function was a quick fix that was intended to allow for off-rotameric states without sacrificing speed and accuracy. Our quick fix may be the cause of overpacking, and possibly the reason for zero hits in this library. Direct inspection of the model instead of automatic design might have identified I152A as a possible mutation to compensate for A163I. The other two buried positions in the target are either conserved (I161I) or conservative (I167V), thus they are not likely to be the source of the problem, attributing the sparce hit rate to A163I and the tight packing around it.

2.2. LOO8-NS1#2 high complexity dengue biosensor library

To avoid the crowding caused by A163I invariant mutation in the first library, a second library was constructed, this one with A163V. The target sequence for this library was YGFGVFTTNIWLR, (residues 934-946 of dengue Type 2 polyprotein) also from NS1, replacing strand 8 sequence 159-NGIKANFKIRHNV-171. Two choices were allowed for each of 38 positions, the computationally selected choice and the wild-type residue. There were 13 designed positions in the core. No fluorescent colonies were found. The following domino series of mutations may have led to the absence of hits in this library (Figure 2). A steric interaction with the target peptide residue V163 forces the selection of Ala from Q183[QA]. This in turn leads to destabilization of Q69, forcing selection of Lys from Q69[QK]. Although K69 forms a favorable buried salt bridge with E222 in the native state, it would likely disrupt E222's role in catalyzing the cyclization of backbone atoms during chromophore formation[60]. This "*safety*" option, in

which the wild-type residue was always in the design palette, provides no safety when the side chain choices are highly covariant, as is the case for the GFP core. In a domino series, initial design choices force other choices, and the resulting design solutions may be too few to find in a screen. In short, the library complexity cannot be too big, or the solution will not be found, and of course it cannot be too small or a solution may not be present.

2.3. LOO11-DMG full-length, low complexity, dengue biosensor library

Acting on the lack of hits in screening high complexity libraries LOO8-NS1#1 and #2, we designed a low-complexity library. In this library the 12-residue, hydrophobic GFP strand 11 sequence 216-DHMVLLEFVTAA-227 was replaced by the NS1 sequence DMGYWIESALND (residues 973-984 of dengue Type 2 polyprotein). In all, 37 positions were designed, with 28 invariant mutations and 9 variable positions in the *native*-format library. The complexity was only 2048 sequences. But LOO11-DMG (also known as LOO11-NS1#5) was abandoned owing to poor expression and therefore no selectable fluorescent phenotype. Poor expression is often equated with incomplete folding. If so, overcrowding in the core may be the culprit. Specifically, the target invariant mutation L220W creates tight interactions with neighboring variable positions F46[IL] and L207[FY].

2.4. LOO8-HIV full-length, low complexity, HIV biosensor library

In parallel with the dengue biosensor libraries, we made biosensors against other viruses. In this library strand 8 sequence 158-NGIKANFTVRHNV-170 is replaced with human immunodeficiency virus 1 envelope protein sequence NGTKGDFTNGNST (residues 417-429 of AEQ75975.1). Six of 13 residues are conserved, including buried side chain F164. As such this biosensor target looked like an easy win, but after computationally designing 28 positions, narrowing 21 positions to one amino acid each, and leaving just seven of them as variable in the

library, no fluorescent colonies were found in the plate screen. The most difficult target position to accommodate and the one most likely position to be responsible for the lack of hits was V167N, which (when combined with H169N) places two asparagines into the hydrophobic core. The N167 and N169 hydrogen bond to each other, but the remaining unsatisfied hydrogen bonding groups extract an energetic cost equivalent of about four bound waters. H-bonding partners could have been designed at neighboring core positions 141 and 145, but these would be non-polar-to-polar substitutions and would have sacrificed stabilizing hydrophobic interactions, therefore they were not selected by Rosetta. Additionally, buried non-polar-to-polar I161T was left without a H-bond partner other than a buried water. No obvious overpacking issues were discovered by inspection of the structure. The lesson learned in this case was that unsatisfied hydrogen bond donors and acceptors must be kept to a minimum. Burying too many polar atoms creates a no-win situation for the design algorithm.

2.5. LOO8-NS1#3 small piecemeal library

Having found zero hits for both high and low complexity libraries when the target is full-length (13 residues in this case), we attempted "*piecemeal*" design. We narrowed our sights to the N-terminal two-residue fragment YG of the LOO8-NS1#2 target. Piecemeal design *in silico* reduced the computational cost of designing a biosensor library by breaking the problem up into small pieces, which would then be combined in a second round, and so on until all pieces are coalesced[16]. Focusing on YG of the LOO8-NS1#2 target to make the LOO8-NS1#3 library represents the first round of piecemeal screening. In this library, 10 colonies out of 65 were fluorescent in the *native* format (our most stable format). The predominance of dark colonies was surprising because the design palette was believed to be very conservative, but when one considers

in hindsight that the folding pathway and protein solubility are also being selected, a low success rate is less surprising.

2.6. LOO8-NS1#4 small piecemeal library

The LOO8-NS1#4 library expression was derived from the sequences of all ten hits from LOO8-NS1#3. The new library was assembled and was screened in the LII format, giving five fluorescent colonies. These results confirmed our expectations that reducing the search space leads to success in library screening and underscores the need for the piecemeal approach.

2.7. LOO7-EDIII high complexity, large piecemeal, dengue biosensor library

Having found that library screening succeeded when the target was divided into small pieces, we explored *piecemeal* design with larger pieces. In these two libraries, strand 7 sequence 145-FNSHNVYIT-153 is replaced by dengue serotype 4 E-glycoprotein (GenBank: QXT92385) sequence NSVTNIELE. The target was split into an N-terminal 4 residue (NSVT), and C-terminal 5-residue (NIELE), pieces for *native* format screening. Table 1 shows the combined libraries. No fluorescent colonies were observed for the N-terminal fragment, but 2 fluorescent colonies were isolated for the C-terminal fragment. To create the LII-format library for the full-length target based on the results of the *native* screens, wild-type residues were added to the palette for designed positions around the N-terminal fragment. The LII-format screening produced several (>4) fluorescent colonies. Sequencing the top 4 brightest colonies produced the solutions highlighted in Table 1. A reasonably high hit rate in the LII format was attained by the large piecemeal approach with a high complexity library, helped perhaps by the fact that the target has only three buried side chains and two of those are conservative changes (V150I, I152L). The third buried target mutation (F145N) places a polar Asn residue in the core. Around N145, Rosetta selected

polar residues and longer side chains, filling the space left by the F->N mutation and satisfying hydrogen bonding of the Asn.

However, screening the same library in the LOO format produced no fluorescent colonies. The essential difference between the LII and LOO formats that explains the lack of hits is the increased entropic barrier in the LOO format. Any destabilizing interaction located in the target interface would have manifested more in the LOO screen, because the free energy of bringing the two parts together adds a barrier to folding and binding. Based on inspection, the most likely destabilizing effect is the target mutation V150I, which, despite being conservative, places the extra methyl group in a tight place between the chromophore and immovable backbone atoms when the isoleucine is in its energetically preferred rotamer. No other rotamer for I150 was selected by Rosetta, even though good second-best rotamer are possible simply by selecting a shorter side chain such as V or A at residue L201. Since only the tightly packed arrangement was chosen, the result represents a design flaw, and it is traceable to the undervaluing of internal spaces by Rosetta's *talaris2013* energy function. Classical force fields do not reward extra space between side chains; indeed, it would be penalized, but entropic considerations suggest that a loosely packed core might be more stable than a tightly packed one. As it is, the modeled target peptide is forced to lose entropy upon binding, creating an activation barrier and possibly slower binding, or slower folding if the entropy loss happens at the rate limiting step. Overpacking in the core, at the peptide/biosensor interface, a design flaw, is the most likely reason this library had no fluorescent colonies.

2.8. LOO7-HA5 recalculated library, medium complexity

We concluded that overpacking in the core resulted in a library of beta barrels that did not close[4]. Therefore a new library LOO7-HA5 was calculated employing rational design, as

described earlier. The library was screened in the *native* format, then rescreened in the LOO format. The results were an improvement over LOO7-HA4 in terms of folding and function. Previously, LOO7-HA4 showed some specific binding to an influenza hemagglutinin peptide, but aggregation and non-closure of the beta barrel led to a negative relative quantum yield (QY) upon binding.

In the LOO7-HA5 library, GFP strand 7 142-EYNFNSHNVYITAD-155 is replaced with influenza hemagglutinin sequence KSSWSSHEVSLGVS (Figure 3). This represents a shift in the hemagglutinin sequence, with corresponding shifts in the LOO7-GFP termini positions. The new site placement includes 145, a buried hydrophobic pocket which can accept the target Trp side chain. The other three inward-facing side chains are either conserved (H18, V150) or conservative (I152L) requiring no changes. T205G was selected by Rosetta to accommodate the *trans* rotamer of W145. Designed mutation H169Y fills the space created by the rotamer shift at 145. Indeed, both T205G and H169Y were found among the fluorescent colonies in our *native*-format library screen. A total of 15 positions were designed, with 4 invariant and 11 variable mutations in the plate screen. One colony fluoresced out of 8 on the plate (transformation efficiency was low in this case). This colony, called ES1 was extensively studied. In ES1, 8 of the 15 designed positions were mutations and 7 were conserved.

2.9. PCR amplification bias not found

We hypothesized that amplification of the sequence library after assembly PCR with degenerate oligos might be non-egalitarian, amplifying a random subset. But sequencing colonies from a simplified, amplified library having only a small sequence complexity showed that PCR amplification was egalitarian (i.e. amplified without bias), given the expected proportions of

sequences in a representative sample of colonies. We assumed therefore that mutations would appear in the transformed bacteria in proportion to amount of the codon in question.
